# Editorial: Microbial-mediated induced resistance: interactive effects for improving crop health

**DOI:** 10.3389/fmicb.2025.1614435

**Published:** 2025-05-19

**Authors:** Debasis Mitra, Anju Rani, Edappayil Janeeshma, Bahman Khoshru

**Affiliations:** ^1^Department of Microbiology, Graphic Era (Deemed to be University), Dehradun, Uttarakhand, India; ^2^Department of Botany, MES KEVEEYAM College, Malappuram, Kerala, India; ^3^Soil and Water Research Institute (SWRI), Agricultural Research, Education and Extension Organization (AREEO), Karaj, Iran

**Keywords:** plant induced systemic resistance, plant growth promoting microorganisms, biocontrol, soil health, sustainable agriculture

The global population, currently estimated at ~8.06 billion, is anticipated to increase to 9.80 billion by 2050 and 11.20 billion by 2100, as reported by the United Nations Department of Economic and Social Affairs (Graham, 2017; UN-DESA, 2024). To address this demographic expansion, it is imperative to prepare for the heightened demand for food. Nevertheless, the agricultural sector remains heavily dependent on chemical fertilizers, pesticides, and herbicides (Zhou et al., 2024). These practices have significant environmental repercussions, including a reduction in the diversity of soil microorganisms, which can ultimately impair food production. This challenge is exacerbated by climate change, declining soil health, and other stressors. In this context, microbial-mediated induced resistance (MIR) emerges as a promising area of research in agriculture, investigating the potential of microbes to enhance plant resistance to pathogens (Singh et al., 2024). This approach utilizes certain microorganisms, such as bacteria and fungi, to trigger a systemic response in plants, thereby boosting their disease defense mechanisms (Rabari et al., 2023). The effect of MIR on crop health can be substantial, providing sustainable alternatives to traditional chemical-based disease management techniques (Manzoor et al., 2024). Progressing research into the role of microbes in sustainable agriculture will encourage the adoption of innovative methods that enhance soil health, crop yield, and fertility.

Soil microorganisms play a vital role in facilitating plant nutrient absorption, inducing systemic resistance, and mitigating adverse climatic conditions through plant signaling compounds and cross-talk mechanisms (Ruparelia et al., 2022). The interactions between beneficial symbiotic microorganisms and plant roots, along with other soil microbial interactions, enhance nutrient utilization efficiency and activate plant defense mechanisms, thereby contributing to sustainable agricultural production (Alzate Zuluaga et al., 2024). This Research Topic aims to present the latest insights into plant-soil-microbe interactions, which play a crucial role in microbial-induced resistance (MIR). Jiang et al. reported that plant growth, crop yield, and pest & disease control are enhanced by plant growth-promoting rhizobacteria (PGPR), which are beneficial microorganisms found in close symbiosis with plant roots. In this study, the efficient PGPR strain T1 was isolated and screened from tobacco inter-root soil, and its identity was confirmed through ITS sequencing technology. The soil's physical and chemical characteristics showed significant enhancement, with phosphorus availability rising by 26.26%. Additionally, there was a marked increase in the activity of essential soil enzymes like sucrase, catalase, and urease, reflecting improved soil health and fertility. This research offers valuable insights for the creation of innovative microbial fertilizers and presents strategies for the sustainable advancement of modern agriculture. The study of Zhu et al. revealed that bacterial wilt, caused by *Ralstonia solanacearum*, significantly hinders the healthy development of tomato seedlings. This study explored the use of biocontrol microbes to combat tomato bacterial wilt, focusing on the behavior of the *Enterobacter hormaechei* Rs-5 and *Bacillus subtilis* SL-44 composite microbial agent (EB) in the rhizosphere soil. The study also evaluated its effects on the soil's microbial community and the growth of tomato plants. EB was found to lower the incidence of tomato bacterial wilt from 77.78 to 22.22% and markedly enhance the biomass, physicochemical properties, and nutrient content of the rhizosphere soil in tomato seedlings. Additionally, there was an increase in the relative abundance of beneficial bacteria such as *Massilia, Pseudomonas, Bacillus*, and *Enterobacter*, along with an improvement in the diversity of the fungal community. Similarly, Manzar et al. examined 260 isolates of *Trichoderma* species, identifying the primary ones as *Trichoderma koningiopsis, T. asperellum, T. caribbaeum* var. caribbaeum, *T. lixii, T. brevicompactum, T. atroviride*, and *T. erinaceum*. Among these, 9% demonstrated significant potential for biocontrol and enhancing crop growth. The use of the effective *Trichoderma* strain TR11 for seed biopriming led to a reduction in the maydis leaf blight (MLB) disease index to 32.92 % and improved growth-promoting characteristics in maize. Additionally, treatments with the TR11 isolate resulted in a 2.5 to 4.2-fold increase in defensive enzyme activities and greater lignification following pathogen inoculation, suggesting bolstered plant defense responses. According to Manjunatha et al., bacterial blight in pomegranates, caused by *Xanthomonas citri* pv. punicae (Xcp), is a highly destructive disease that results in significant financial losses in pomegranate farming. The use of endophytes such as *Bacillus haynesii, B. tequilensis*, and *B. subtilis* for controlling this blight led to a 47–68% decrease in the disease index, which is notably more effective than the reduction achieved by the chemical immune modulator (2-bromo-2-nitro-1, 3-propanediol) that is currently recommended for managing the blight. Research conducted by Hima Parvathy et al. indicates that black pepper (*Piper nigrum* L.) is susceptible to foot rot caused by the soil-borne oomycete pathogen *Phytophthora capsici*. The investigation highlighted compositional variations in the rhizobiome of two Piper species, with *P. colubrinum* exhibiting greater diversity and a higher number of differentially abundant genera. Predictive functional profiling of the *P. colubrinum* rhizobiome identified a significant enrichment of functional gene orthologs (KOs), notably chemotaxis proteins, osmoprotectants, and various transport systems that contribute to pathogen resistance. A study by Tomer et al. examined the phosphate solubilizing abilities of *Lysinibacillus macroides* ST-30, *Pseudomonas pelleroniana* N-26, and Bacillus cereus ST-6 in relation to chickpea cultivation in the Tarai region of Uttarakhand. The consistent presence of these inoculated P solubilizers throughout the experiment indicates their capability to compete with native microflora and maintain a good shelf life under field conditions, supporting their potential use as commercial fertilizers in the future. Lee et al. emphasized the role of *Bacillus megaterium* strains CACC109 and CACC119, isolated from a ginseng field, in enhancing drought stress tolerance through plant growth-promoting activities. They explored these mechanisms by assessing the strains' impact on rice growth and stress resilience using *in vitro* assays, pot experiments, and physiological and molecular analyses. The application of CACC109 and CACC119 led to increased expression of genes related to antioxidants and drought response. These strains show promise as biostimulants for boosting plant growth and providing resistance to abiotic stresses in crop production. Bao et al. found that an increase in soil Actinobacteria and Firmicutes, or a decrease in Gemmatimonadetes and Myxococcota, could create a favorable environment for the sustainable growth of medicinal plant crops in salinized soil ecosystems. Lin et al. discovered that dazomet, a soil fumigant, effectively controls soil-borne pathogens and boosts levels of total N, P, K, available ammonia nitrogen, P and K in the soil. Its fumigation also increased the relative-abundance of bacteria involved in the biosynthesis of secondary metabolites, while decreasing the relative-abundance of pathogenic fungi and reducing the incidence of soil- borne diseases. Choudaker et al. highlighted the success of using microbial antagonists, especially the *B. subtilis* DTBS-5, in controlling wheat PM through biocontrol, induced resistance, and improved plant growth, presenting a sustainable alternative to chemical methods. Garg et al. discussed that plant biostimulants comprise biomolecules like lipids, carbohydrates, proteins, and other secondary metabolites associated with specific nitrogen-containing compounds, terpenes, and benzene ring-conjugated compounds. These secondary metabolites, being crucial precursors, require extensive study for precise calculations of biochemical reactions occurring inside and outside the synthesized living cell. This review underscores sequencing techniques as a basis for generating opportunities in agricultural sustainability. Zeng et al. study demonstrated a notable rise in the presence of beneficial bacteria in the rhizosphere soil of *Achyranthes bidentata* when subjected to extended monoculture, as indicated by bioinformatics analysis. The functional analysis revealed a variety of plant growth-promoting characteristics among these bacteria, such as the production of indole-3-acetic acid in the range of 68.010–73.250 mg/L, abilities to solubilize P and K, and antagonistic effects against pathogenic fungi (21.540–50.810%). Malik et al. investigated the effects of biosynthesized silver nanoparticles (AgNPs) derived from *Rhizoctonia solani* and *Cladosporium cladosporioides* through a green synthesis method, assessing their antifungal activity against various pathogenic fungi. A concentration of 15 mg/mL of these AgNPs exhibited strong inhibitory effects on all tested fungal pathogens. Consequently, the findings strongly indicate that silver nanoparticles could play a significant role in managing different plant diseases caused by fungi.

In conclusion, we believe that this Research Topic on “*Microbial-mediated induced resistance: interactive effects for improving crop health*” will provide significant insights into the recent advancements and benefits of using PGPR and biostimulants to achieve sustainable agricultural production. Additionally, it will highlight the role of microbial inoculants in enhancing crop yields while maintaining soil health.

## References

[B1] Alzate Zuluaga M. Y. Fattorini R. Cesco S. Pii Y. (2024). Plant-microbe interactions in the rhizosphere for smarter and more sustainable crop fertilization: the case of PGPR-based biofertilizers. Front. Microbiol. 15:1440978. 10.3389/fmicb.2024.1440978 39176279 PMC11338843

[B2] Graham L. (2017). UN raises world population forecast to 9.8 billion people by 2050 due to rapid growth in Africa. CNBC. Available online at: https://www.cnbc.com/2017/06/22/un-raises-world-population-forecast-to-9-point-8-billion-people-by-2050.html (accessed January 3, 2019).

[B3] Manzoor S. Nabi S. U. Rather T. R. Gani G. Mir Z. A. Wani A. W.. (2024). Advancing crop disease resistance through genome editing: a promising approach for enhancing agricultural production. Front. Genome Ed. 6:1399051. 10.3389/fgeed.2024.1399051 38988891 PMC11234172

[B4] Rabari A. Ruparelia J. Jha C. K. Sayyed R. Z. Mitra D. Priyadarshini A.. (2023). Articulating beneficial rhizobacteria-mediated plant defenses through induced systemic resistance: a review. Pedosphere 33,556–566. 10.1016/j.pedsph.2022.10.003

[B5] Ruparelia J. Rabari A. Mitra D. Panneerselvam P. Das-Mohapatra P. K. Jha C. K. (2022). Efficient applications of bacterial secondary metabolites for management of biotic stress in plants. Plant Stress 6:100125. 10.1016/j.stress.2022.100125

[B6] Singh D. P. Maurya S. Satnami L. Prabha R. Sarma B. K. Rai N. (2024). Roots of resistance: unraveling microbiome-driven plant immunity. Plant Stress 14:100661. 10.1016/j.stress.2024.100661

[B7] UN-DESA (2024). The World Population Prospects: The 2017 Revision, published by the UN Department of Economic and Social Affairs. Available online at: https://www.un.org/en/desa/world-population-projected-reach-98-billion-2050-and-112-billion-2100 (accessed 18 April, 2025)

[B8] Zhou W. Li M. Achal V. (2024). A comprehensive review on environmental and human health impacts of chemical pesticide usage. Emerg. Contam. 11:100410. 10.1016/j.emcon.2024.100410

